# The Effects of Price on Alcohol Consumption and Alcohol-Related Problems

**Published:** 2002

**Authors:** Frank J. Chaloupka, Michael Grossman, Henry Saffer

**Affiliations:** Frank J. Chaloupka, Ph.D., is a professor in the Department of Economics and director of ImpacTeen: A Policy Research Partnership to Reduce Youth Substance Abuse at the Health Research and Policy Centers, University of Illinois at Chicago, Chicago, Illinois. He is also a research associate in the Health Economics Program, National Bureau of Economic Research (NBER), New York. Michael Grossman, Ph.D., is a research associate and director of the Health Economics Program, NBER, and Distinguished Professor of Economics at the City University of New York Graduate Center, New York. Henry Saffer, Ph.D., is a research associate in the Health Economics Program, NBER, New York, and professor of economics at Kean University of New Jersey, Union, New Jersey

**Keywords:** alcohol or other drug (AOD) price, economic theory of AOD use (AODU), elasticity of demand, underage drinking, minimum drinking age, drinking and driving, AOD related (AODR) accident mortality, AOD availability, AODR injury, AODR crime, AODR violence, sales and excise tax

## Abstract

The most fundamental law of economics links the price of a product to the demand for that product. Accordingly, increases in the monetary price of alcohol (i.e., through tax increases) would be expected to lower alcohol consumption and its adverse consequences. Studies investigating such a relationship found that alcohol prices were one factor influencing alcohol consumption among youth and young adults. Other studies determined that increases in the total price of alcohol can reduce drinking and driving and its consequences among all age groups; lower the frequency of diseases, injuries, and deaths related to alcohol use and abuse; and reduce alcohol-related violence and other crime.

Since the early 1980s, a growing number of economists have examined the impact of the price of alcoholic beverages on alcohol consumption. Other studies have evaluated the effects of price on various outcomes related to alcohol consumption, including nonfatal and fatal motor vehicle crashes and other injuries, liver cirrhosis and other alcohol-related mortality, and violence and other crime. This research, which has used a wide variety of data, generally has concluded that increases in the prices of alcoholic beverages lead to reductions in drinking and heavy drinking as well as in the consequences of alcohol use and abuse. This conclusion concurs with a fundamental law of economics called the downward sloping demand curve, which states that as the price of a product rises, the quantity demanded of that product falls. Since the price of alcohol can be manipulated through excise tax policies, the findings regarding the relationship between alcohol price and alcohol consumption clearly are relevant for policy-makers interested in reducing alcohol consumption and its adverse consequences. Indeed, Federal, State, and local governments have implemented many policies to combat alcohol abuse in the past two decades (see sidebar).

**Policies Affecting the Price of Alcohol and Alcohol Consumption**

The major policy element of U.S. programs to deter teenage and young adult drinking has been to increase State minimum legal drinking ages (MLDAs). This trend began in 1976 with the increase in the MLDA in Minnesota from 18 to 19 years of age. An additional 27 States had increased their MLDAs by the time Congress passed the Federal Uniform Drinking Age Act of 1984. This Act pressured all States into raising the MLDA to 21 years by withholding part of their Federal highway funding if they failed to comply. After an unsuccessful challenge to the law’s constitutionality, all States raised their MLDAs to 21 years by July 1988.^1^ In addition, several States have recently adopted laws targeting underage drinking drivers by making it *per se* illegal to drive either with blood alcohol concentrations (BACs) well below those considered the legal limit of intoxication in adults (i.e., 0.08 to 0.1 percent) or, in some States, with any measurable BACs. These measures have made it more difficult for youths to obtain alcohol and have increased the expected legal costs of drinking by imposing fines for the possession of alcohol and for drinking and driving by underage people.

Other policies in the campaign to discourage alcohol abuse have targeted all drinkers, and some measures have targeted abusive drinkers. For example, Public Law 100–690 mandated that, beginning in November 1989, a label warning of the dangers of drinking and driving, alcohol consumption during pregnancy, and other (unspecified) health consequences of drinking appear on all alcoholic beverage containers. By raising awareness of potential health consequences of drinking, such warning labels can increase consumers’ perceptions of the costs associated with drinking alcoholic beverages.

Similarly, the Alcohol Traffic Safety Act of 1983 encouraged States to enact hundreds of new and stronger laws related to driving under the influence of alcohol (DUI). For example, these measures eased the standards required for arresting and convicting drunk drivers, imposed more severe and certain penalties upon conviction for drunk driving, and increased the allocation of resources for apprehending drunk drivers. Again, such increases in the probabilities of arrest and conviction for drunk driving as well as in the penalties upon conviction raised the expected legal costs of drinking and driving.

Many States and localities also have adopted policies that raise the time costs associated with obtaining alcoholic beverages or otherwise reduce alcohol availability for all drinkers. For example, these policies include regulations that limit the places and/or times where alcohol can be sold, restrict or ban “happy hours,” require server training and/or licensing, and hold servers liable for the harmful actions related to the excessive drinking of those they are serving.

One policy that has only rarely been used, however, is an increase in the monetary price of alcohol, which could be achieved by raising taxes on alcoholic beverages. During the past 50 years, Federal, State, and local taxes on alcohol have been raised only modestly and infrequently and almost always with the intent of increasing revenues rather than discouraging alcohol use and abuse. For example, Title XI of the Omnibus Budget Reconciliation Act of 1990 increased Federal excise tax rates on beer and wine for the first time since November 1951 and the tax on distilled spirits for only the second time during this period.^2^ This act doubled the tax on beer from 16 cents to 32 cents per six-pack; raised the tax on wine nearly sevenfold, from just over 3 cents to about 21 cents per 750-ml bottle; and increased the tax on distilled spirits from $2 to $2.16 per fifth of 80-proof alcohol.

Although some evidence suggests that Congress may have been motivated by the health promotion aspects of higher alcoholic-beverage taxes (Cook and Moore 1993), these increases fell well short of those recommended by several public health organizations (Godfrey 1990). The increases also left the tax rates well below the 25 cents per ounce of pure alcohol in any beverage that initially had been proposed by the first Bush Administration (Godfrey 1990). Thus, the Federal excise tax rates resulting from the 1991 tax increase are approximately 10 cents, 7 cents, and 21 cents per ounce of pure alcohol for beer, wine, and distilled spirits, respectively. Furthermore, these tax increases failed to offset inflation since 1951. For example, the tax on distilled spirits would have needed to increase to approximately $8.80 per fifth of 80-proof liquor (i.e., approximately fourfold higher) to have the same real value as this tax had in 1951. Similarly, the tax on beer would have needed to increase more than fivefold to 84 cents per six-pack to offset inflation. Only the increase in the wine tax was large enough to offset the effects of inflation since 1951.^3^

The potential effects of greater increases in the monetary price of alcohol are explored in the feature article.

— Frank J. Chaloupka, Michael Grossman, Henry Saffer

1So-called grandfather clauses in some States kept the effective age below 21 until mid-1989.2Excise taxes differ from sales taxes in that they are a flat tax amount based on the quantity purchased rather than a certain percentage of the purchase price. Generally, excise taxes are not paid directly by consumers but are passed on to consumers in the form of higher retail prices. In contrast, consumers pay a sales tax directly when they make a purchase.3These computations are based on Bureau of Labor Statistics data from various years.

ReferencesCookPJMooreMJTaxation of alcoholic beveragesHiltonMEBlossGEconomics and the Prevention of Alcohol-Related ProblemsNIAAA Research Monograph No. 25NIH Pub. No. 93–3513Bethesda, MDNational Institute on Alcohol Abuse and Alcoholism19933358GodfreyJAlcohol taxes: From the whiskey rebellion to the 1990 budget summitTax Notes4821371990

One policy that has largely been ignored, however, is an increase in the monetary price of alcohol, which could be achieved by raising taxes on alcoholic beverages. At least in part as a result of this stability of Federal, State, and local alcoholic beverage taxes, the real prices of alcoholic beverages (i.e., the prices after accounting for the effects of inflation) have declined significantly over time. For example, between 1975 and 1990, the real price of distilled spirits fell by 32 percent, the real price of wine fell by 28 percent, and the real price of beer fell by 20 percent.^1^ A Federal tax increase in 1991 only temporarily reversed this trend. If alcohol use and abuse are sensitive to price, as economists have found, however, a decrease in the real value of alcoholic beverage taxes and, consequently, prices will exacerbate the problems associated with alcohol use and abuse. Governments may be reluctant to increase taxes to discourage alcohol abuse, however, because the increased taxes raise prices not only for alcohol abusers but also for light and moderate drinkers who do not abuse alcohol and therefore do not need to be discouraged from drinking. (For more detailed discussion of the appropriate level of alcohol taxation in this context, see Pogue and Sgontz 1989; Saffer and Chaloupka 1994.)

This article reviews studies that have analyzed the effects of price increases on alcohol consumption and its adverse consequences. After discussing some analytical considerations, the article focuses on the effects of alcohol prices and taxes on consumption by youths and young adults. It then considers the impacts of prices and taxes on indicators of alcohol abuse, such as motor vehicle crashes, health effects, and violence and other crime, among drinkers of all ages, including youths and young adults. The studies discussed in this article capitalize on the substantial variation in alcohol prices across the United States that exists primarily as a result of the vastly differing State excise tax rates on alcoholic beverages. Most of the studies employ the price of beer or the State excise tax on beer as a measure of the cost of alcohol because beer is the beverage of choice among Americans, particularly among youths.

## Analytical Considerations

Economic studies of alcohol demand focus mainly on the effects of price on alcohol consumption. To describe the sensitivity of consumption to changes in monetary price, economists frequently refer to the price elasticity of demand^2^ (i.e., the percentage change in consumption resulting from a 1-percent increase in price). For example, a price elasticity of alcohol demand of −0.5 means that a 1-percent increase in price would reduce alcohol consumption by 0.5 percent (or a 10-percent increase in price would reduce consumption by 5 percent). An extensive review of the economic literature on alcohol demand concluded that based on studies using aggregate data (i.e., data that report the amount of alcohol consumed by large groups of people), the price elasticities of demand for beer, wine, and distilled spirits are −0.3, −1.0, and −1.5, respectively (Leung and Phelps 1993).^3^ These estimates suggest that beer consumption is relatively insensitive to price changes, whereas demand for wine and distilled spirits is very responsive to price.

Analyses using individual-level data (i.e., data that report the amount of alcohol consumed by specific persons) suggest that alcohol demand may be even more responsive to price than these estimates indicate, possibly because this approach can obtain differential price responses among respondents of different age groups (Leung and Phelps 1993). More recent studies have confirmed the price responsiveness of alcohol consumption (Nelson 1999; Kenkel 1993, 1996; Manning et al. 1995).

One consideration that must be kept in mind when interpreting price effects such as those discussed throughout this article is that these effects are not based on natural experiments. For example, no data are available comparing the amounts of alcohol consumed by individuals or groups at different prices, with all other variables held constant. Instead, researchers use cross-sectional data, which measure consumption for individuals or groups at a given moment in time, or time series of such cross-sectional analyses from more than 1 year. And although investigators in these studies attempt to control for as many confounding variables (i.e., variables that may be correlated with price and consumption) as possible, these efforts can never be complete. These caveats place limits on the ability to infer cause-and-effect relationships from the study findings.

Another consideration when analyzing price effects on alcohol consumption is the potentially addictive nature of alcohol. Next to cigarette smoking, excessive drinking is the most common example of legally consuming an addictive substance. However, alcohol and tobacco are linked to adverse health outcomes and to addiction in different ways. For example, overwhelming evidence indicates that any level of smoking has detrimental health effects. Furthermore, a large proportion of smokers become addicted to nicotine and therefore smoke a substantial quantity of cigarettes each day. Accordingly, researchers can usually focus their analyses on whether and how much a person smokes because these measures are highly correlated with the smoking-related costs of interest.

With alcohol, however, the situation is more complex. Many people regularly consume small quantities of alcohol without becoming addicted. Furthermore, most people who drink alcohol do not harm themselves or others; indeed, moderate alcohol consumption has been shown to lower the risk of coronary heart disease in men (Camargo et al. 1997). The adverse effects of alcohol, such as liver cirrhosis, drunk-driving crashes, workplace injuries, and various forms of violent behavior, primarily result from excessive consumption (regardless of whether the person is actually addicted to alcohol). Researchers must consider these complex interactions (e.g., specific drinking patterns) when exploring the relationship between alcohol price and alcohol consumption or alcohol-related adverse effects.

## Drinking by Youths and Young Adults

Much of the alcohol-related economic research considers alcohol consumption by all segments of the population. Nevertheless, it is crucial to focus on the price sensitivity of youth and young-adult drinking and heavy drinking because the incidence of alcohol-related problems, particularly drinking and driving, is disproportionately high among these age groups. Fatal motor vehicle crashes are the leading cause of death of people under the age of 35, and alcohol is involved in more than one-half of these fatal crashes. In 1995, fatalities per car miles of travel of people between the ages of 16 and 24 were more than twice as large as those of people ages 25 and over (Dee and Evans 2001). Moreover, abuse of and dependence on alcohol are highest among people between the ages of 18 and 29 (Grant et al. 1991). Finally, it is important to focus on the young because alcohol abuse in adolescence appears to be associated with alcohol abuse in later life (Rachal et al. 1980). Consequently, policies to curb alcohol abuse by youths and young adults might be the most effective means to curb it in all segments of the population.

Two studies estimated the effects of price on alcohol use by youths ages 16 to 21 using data from the National Health and Nutrition Examination Surveys (Grossman et al. 1987; Coate and Grossman 1988). The data were collected in two cycles of surveys conducted from 1971 to 1975 and from 1976 to 1980, respectively. Both studies concluded that beer consumption is inversely related to both the monetary price of beer and the State minimum legal drinking age (MLDA). The studies also evaluated whether the effects of price differ according to the youths’ consumption patterns. To this end, the investigators classified the youths into infrequent drinkers who consumed beer less than once per week, fairly frequent drinkers who consumed beer one to three times per week, and frequent drinkers who consumed beer four to seven times per week. These analyses found that higher prices and MLDAs reduced not only the fraction of youths who drank beer infrequently but that the fractions of youths who consumed beer fairly frequently and frequently declined more in both absolute and percentage terms than did the fraction of infrequent drinkers when prices rose. The investigators also conducted similar analyses for different drinking levels among the youth, including light drinkers who consumed one or two cans of beer, fairly heavy drinkers who consumed three to five cans, and heavy drinkers who consumed six or more cans on a typical drinking day. Again, the fractions of fairly heavy and heavy drinkers declined more in both absolute and percentage terms than did the fraction of light drinkers in response to price increases.

Laixuthai and Chaloupka (1993) updated this research using data from the 1982 and 1989 surveys of high school seniors conducted by the University of Michigan’s Institute for Social Research as part of the Monitoring the Future project. The analyses used three different measures of alcohol consumption that were constructed from the survey data, as follows:

- Drinking frequency in the past year, which classified youths as frequent drinkers (more than 30 drinking occasions in the past year), fairly frequent drinkers (10 to 30 drinking occasions), infrequent drinkers (1 to 9 drinking occasions), or abstainers (no drinking in the past year)
- Drinking frequency in the past month, which was structured similarly
- The presence of at least one binge-drinking occasion (i.e., consumption of five or more drinks on one occasion) in the 2 weeks prior to the survey. These binge-drinking occasions, which serve as an indicator of heavy drinking, are most likely to have negative consequences and, therefore, are of most concern to policymakers.

The researchers analyzed the 1982 and 1989 samples separately to examine potential changes in the price sensitivity of youth alcohol use resulting from the introduction of a uniform MLDA of 21 years in all States. For both years, higher beer excise taxes significantly reduced both the frequency of youth drinking and the probability of heavy drinking. As with the studies by Grossman and colleagues (1987) and Coate and Grossman (1988), the estimates implied that a tax increase would reduce the fractions of frequent and fairly frequent young drinkers to a greater extent than the fraction of infrequent drinkers. More interestingly, Laixuthai and Chaloupka (1993) also found that the price sensitivity of youth drinking fell after the MLDA of 21 years was enacted in all States. For example, the investigators estimated that an increase in the Federal beer tax offsetting the effect of inflation since 1951 would have reduced the probability of having any binge-drinking episodes by 18.4 percent in 1982 but only by 6.5 percent in 1989.

Laixuthai and Chaloupka (1993) attributed this change in the price sensitivity of youth drinking to the change in the full price of drinking resulting from the higher MLDAs in 1989. For a youth, the full price of consuming alcohol can be thought of as the monetary price of alcohol plus the indirect costs of illegal drinking. These indirect costs include such legal obstacles as the MLDA, the time spent obtaining alcohol, and the money and time spent obtaining false identification. When the average MLDA and, consequently, the associated indirect costs of alcohol, are relatively low (as in the 1982 sample), a given increase in alcohol taxes will have a relatively large impact on the full price of alcohol and thus on consumption. Conversely, when the average MLDA and the associated indirect costs of alcohol are high (as in the 1989 sample), a similar increase in alcohol taxes will have a relatively small impact on the full price of alcohol and on consumption. Accordingly, high school seniors in 1989, who faced higher indirect costs of obtaining alcohol than their 1982 counterparts, responded less to changes in the monetary costs.

Chaloupka and Wechsler (1996) examined the effects of various factors on drinking and binge drinking among students in U.S. colleges and universities. The analyses were based on data from the 1993 Harvard College Alcohol Survey, which included a nationally representative sample of 17,592 students enrolled at 140 U.S. 4-year colleges and universities. The alcohol-related factors evaluated included beer prices, alcohol availability (i.e., the presence of an on-campus bar and the number of alcohol outlets within 1 mile of the campus), and policies related to driving under the influence (DUI) (i.e., a State-level index reflecting the restrictiveness of the State’s drunk-driving laws targeting youths).

The investigators estimated the potential results of a policy that would have equated the tax on the alcohol in beer to the tax on the alcohol in distilled spirits in 1951 and adjusted the tax for the rate of inflation since 1951. Such an increase would have resulted in a more than tenfold increase in the tax. The results implied that such a policy would have reduced the number of underage college women who drank in the past year by about 15 percent and the number of underage and older college women engaging in any binge drinking by roughly 20 percent. In contrast to these statistically significant negative effects of price on underage drinking and binge drinking by female students, no such effect was found for male students.

The insignificant effects of price on drinking among male college students and the relatively small effects for female college students likely result, at least in part, from errors in the measure of price used (Chaloupka and Wechsler 1996). Researchers generally use average local retail prices as a measure of the monetary price of alcohol, thereby neglecting alcohol consumption that occurs at parties or other occasions at which the drinker does not pay retail price for the alcohol. Such errors are a general problem in econometric studies of alcohol demand using individual-level data but likely are more significant when studying college students, for whom average local retail prices may not be a good proxy for the prices paid by the student. For example, much of the drinking among college students, particularly binge drinking, takes place at parties where alcohol is available at no charge or at local bars that offer sharply discounted prices to attract college students.

### Studies Accounting for the Addictive Nature of Alcohol

At least for some consumers, the demand for alcoholic beverages may differ from the demands for most other consumer products because of the addictive nature of alcohol. Prior to the work by Becker and Murphy (1988), economic models of addiction assumed myopic behavior in which consumers ignore the future consequences of their current actions. Becker and Murphy (1988) developed a theoretical model that extends the utility-maximizing approach of economics to addictive substances. The consumption of these substances is influenced not only by their utility and the satisfaction they provide but also by acquired tolerance, reinforcement, and withdrawal. The main element of this and other models of addiction is the assumption that an increase in the past consumption of an addictive substance raises the current consumption of that substance. Unlike previous models of addiction, the Becker-Murphy model treats addicts as “farsighted” in the sense that they consider, at least to some extent, the future consequences of their consumption decisions. This assumption implies that a person’s current consumption decisions will respond to changes in the expected future costs of consumption, such as an anticipated increase in price or new information about the health consequences of consumption. Although this assumption may appear to be counterintuitive, it generates a prediction that can be tested using data on alcohol consumption by the same person in 3 or more years. For example, the model predicts that the benefits of consumption this year depend on expected consumption next year. Accordingly, a reduction in next year’s consumption due to an increase in next year’s price should cause this year’s consumption to fall, a prediction that can be readily tested.

The Becker-Murphy model also predicts that the short-term price elasticity, which holds past consumption constant, must be smaller in absolute value than the long-term price elasticity, which allows past consumption to vary.^4^ For example, a price increase in 2001 according to the model would reduce consumption in 2001, with consumption in previous years held constant. Because of the addictive nature of alcohol, the model also predicts that consumption in 2002 and in all future years also would fall. Consequently, the reduction in consumption observed over several years (i.e., in the long term) after the price increase would exceed the reduction observed in 2001 (i.e., in the short term).

Grossman and colleagues (1998) applied the Becker-Murphy model to alcohol consumption by young adults ages 17 to 29 using the longitudinal data from the Monitoring the Future project. Given that the prevalence of alcohol dependence and abuse is highest in this age group (Grant et al. 1991), such an approach accounting for the addictive aspects of alcohol consumption may be more relevant to this sample than to a sample including all age groups. Using data obtained in baseline surveys of high school seniors conducted from 1976 through 1985 and in followup surveys conducted through 1989, the investigators estimated alcohol demand both in the context of the model of addictive behavior and in the context of models that ignore the addictive aspects of consumption.

The study found consistent evidence that increases in the price of alcohol resulting from higher monetary prices significantly reduced the number of alcoholic drinks consumed by young adults in the past year. Moreover, the analyses provided strong evidence that drinking in this age group is addictive in the sense that a strong interdependency existed among past, current, and future alcohol consumption. That is, current drinking decisions depended on past alcohol consumption and influenced future consumption. These findings are generally consistent with studies employing data from the National Longitudinal Survey of Youth to estimate alcohol demand using a model that also accounts for the addictive nature of alcohol consumption.

The finding that drinking by young adults can be considered an addictive behavior has important implications for the effects of price on alcohol consumption. For example, when Grossman and colleagues (1998) used models that ignored the addictive aspects of alcohol consumption to analyze their data, they estimated an average price elasticity of alcohol demand of −0.29. When they used the model accounting for the addictive nature of alcohol, however, the estimated average long-term price elasticity of demand was more than twice as high at −0.65, indicating that price had a much greater influence on alcohol consumption. Moreover, the estimate of the long-term price elasticity of demand was approximately 60 percent higher than the estimate of the short-term elasticity (which, in turn, was almost 40 percent higher than the average estimate derived using nonaddictive models).

Using the estimates derived from models accounting for addiction, Grossman and colleagues (1998) predicted the effects of changes in beer taxes on consumption. For example, the investigators examined the effects of a tax increase that would have matched the taxes on the alcohol in beer to those on the alcohol in distilled spirits in 1951 and then accounted for the rate of inflation since 1951. Such an increase was estimated to have reduced average consumption by more than 40 percent in 1982 and 1983 (the middle years of the sample).

Taken together, these findings on the relationship between price and demand for alcohol have important implications for policies aimed at curbing alcohol use and abuse among youths and young adults. First, this research demonstrates that increases in the price of alcoholic beverages, which could be achieved by raising alcohol taxes, effectively can reduce drinking and heavy drinking. Second, the results demonstrate that the long-run price elasticity of demand when accounting for the addictive aspects of drinking is well above both the short-run elasticity and the elasticity obtained when ignoring addictive aspects. This finding implies that previous estimates of the effects of tax increases on alcohol use among youths and young adults and its consequences significantly understate the benefits of higher taxes. Third, the finding that young adults are farsighted in terms of future alcohol consumption implies that policies that raise the perceived future costs of alcohol use and abuse can significantly reduce current drinking.

### Limitations of the Analyses

As is the case with much social science research, these findings and implications must be qualified because they are not derived from controlled experiments that can definitively establish that a certain factor causes a specific outcome. One can argue that the effects of taxes or prices in the studies just summarized are biased because they do not account for unmeasured determinants of consumption that are correlated with the cost of alcohol. For example, States in which antidrinking sentiment is widespread and alcohol consumption is low may enact high alcohol excise taxes as part of the political process. In this case, the price elasticities that emerge from analyses that only consider price but omit overall drinking sentiment overstate the true influence of price. Conversely, States in which pro-drinking sentiment is widespread (i.e., antidrinking sentiment is weak) and alcohol consumption is high may enact high alcohol taxes because the taxation of alcohol is an attractive source of revenue. In these cases, price elasticities are understated if they are obtained from analyses that omit drinking sentiment.

Using data from the Monitoring the Future surveys of high school seniors conducted between 1977 and 1992, Dee (1999) addressed this issue by comparing the relationship between alcohol consumption and State beer excise taxes among all States over time. In this analysis, Dee included a fixed-effects indicator for each State to control for unmeasured determinants of alcohol consumption and the State’s excise tax on beer.^5^ This analysis found that once the State indicators were held constant, beer excise taxes no longer had a significant negative effect on consumption, suggesting that other, unmeasured factors rather than differences in price account for differences in alcohol consumption.

These conclusions are not definitive, however. For example, Grossman and colleagues (1998) found only a modest reduction in their estimate of the long-term price elasticity of demand (i.e., from −0.65 to −0.54) when controlling for fixed effects. Furthermore, Cook and Moore (2001), who used data on young adults participating in the National Longitudinal Survey of Youth conducted between 1982–1985 and 1988–1989, found that the effect of the State beer tax on drinking participation and binge drinking actually increased significantly in State fixed-effects models.

This discrepancy in findings may stem from the fact that the relative stability of the beer tax makes it highly correlated with other State indicators (e.g., overall drinking sentiments). Accordingly, it is difficult to distinguish the effects of the beer tax and other State fixed effects. Furthermore, as mentioned earlier, State excise taxes are an imperfect measure of the price of alcohol, and biases resulting from measurement errors are exacerbated in fixed-effects models. Thus, although most of the empirical literature supports the conclusion that excise tax increases tend to curtail alcohol consumption and heavy drinking by underage youths and young adults, more research on this important issue is necessary.

## Effects of Price on Consequences of Alcohol Abuse

In addition to examining the effects of the price of alcohol on consumption, numerous economists have studied the impact of price on consequences of alcohol use and abuse. These consequences include fatal and nonfatal motor vehicle crashes and other injuries, liver cirrhosis mortality and other health consequences, and violence and other crime. This section summarizes findings from recent research conducted by the authors of this article as well as from other key studies (for a more detailed review of this literature, see Chaloupka et al. 1998). Because MLDAs and drunk-driving laws play important roles in motor vehicle crashes, their effects as well as those of prices or taxes also are discussed.

### Motor Vehicle Crashes

Saffer and Grossman (1987*a*,*b*) first examined the impact of beer excise taxes and MLDAs on youth fatality rates from motor vehicle crashes. The investigators used State-level fatality rates for youths ages 15 to 17, 18 to 20, and 21 to 24 for the years 1975 through 1981 and controlled for various other factors expected to affect drinking and driving and the probability of fatal crashes. Both studies concluded that increases in beer taxes or MLDAs would significantly reduce youth motor vehicle fatalities. For example, the studies predicted that a policy adjusting the beer tax for the inflation rate since 1951 would have reduced fatalities among 18- to 20-year-old youths by 15 percent. Moreover, a uniform MLDA of 21 years would have lowered youth fatalities by 8 percent between 1975 and 1981.

Chaloupka and colleagues (1993) extended and updated this research by considering the effects of beer taxes, MLDAs, alcohol availability, and all major State-level policies related to drinking and driving on youth and adult motor vehicle fatality rates for the period from 1982 through 1988. The study included numerous drunk-driving policies, as follows:

- Implied consent laws, which presume that a person with a driver’s license agrees to be tested for alcohol and other drugs on request or face license suspension or revocation
- Preliminary breath tests prior to arrest to establish probable cause for a DUI arrest
- No-plea-bargaining provisions, which prohibit a person charged with DUI to plea bargain to reduce the charge to a nonalcohol-related offense, such as reckless driving
- Dram shop laws, which allow those injured by an intoxicated person to bring suit against the person or establishment that served the alcohol
- Administrative *per se* laws, which require the state licensing agency to suspend or revoke a person’s license after a DUI arrest but prior to any court penalty
- Open container laws, which make it illegal to carry open containers of alcoholic beverages in the car
- Mandatory fines, license suspension/revocation, jail sentences, and/or community service upon conviction for drunk driving.

In addition to examining overall fatality rates, the researchers considered two fatality rates that are closely related to drinking and driving:

- The number of drivers killed between 12:00 a.m. and 3:59 a.m., 75 to 90 percent of whom have been estimated to have been drinking (National Highway Traffic Safety Administration 1986)
- The number of drivers with elevated BACs killed in traffic crashes.

The study by Chaloupka and colleagues (1993) concluded that several drunk-driving laws, especially laws associated with relatively severe sanctions, can be effective deterrents to drinking and driving. In particular, the investigators found that whereas existing administrative *per se* license suspensions with relatively weak penalties have little effect on fatality rates, a relatively severe mandatory license suspension of 1 year significantly reduces drunk driving. Severe mandatory minimum fines and license sanctions upon conviction for DUI are also effective deterrents, although somewhat less effective than more immediate administrative penalties that can be imposed without court proceedings. In addition, both preliminary breath test laws, which raise the probability of arrest for DUI, and no-plea-bargaining provisions, which raise the expected penalties, deter drinking and driving. Reduced availability, resulting from both local prohibitions on alcohol sales and higher MLDAs, also can reduce drinking and driving, although the MLDA effects were limited to youths and young adults.

Chaloupka and colleagues (1993) also concluded that higher beer excise taxes are among the most effective means for reducing drinking and driving in all segments of the population. For example, between 1982 and 1988, a policy adjusting the Federal beer tax for the inflation rate since 1951 would have reduced total fatalities by 11.5 percent and fatalities among 18- to 20-year-olds by 32.1 percent.

More recent research using both aggregate and individual-level data similarly has concluded that increases in beer taxes and MLDAs, as well as strong laws related to drinking and driving, can reduce self-reported drinking and driving and involvement in nonfatal traffic crashes. For example, a comprehensive study using aggregate data for the period from 1982 through 1988 found consistent evidence that higher beer taxes significantly reduce motor vehicle crash fatalities in a variety of models that account for potential omitted variables biases (Ruhm 1996).^6^ These findings are notable because most of the models used included State-fixed effects. Another study based on self-reported data on drinking and driving obtained in the 1985 National Health Interview Survey estimated that a 10-percent increase in the price of alcoholic beverages would reduce the probability of drinking and driving by about 7.4 percent for men and 8.1 percent for women (Kenkel 1993). Even larger reductions of 12.6 percent among men and 21.1 percent among women would occur among people ages 21 years and younger. A study using self-reported data on involvement in traffic crashes obtained during the 1982 and 1989 Monitoring the Future surveys concluded that a policy adjusting the Federal beer tax for the inflation rate since 1951 would reduce the probability of nonfatal traffic crashes by almost 6 percent for both men and women (Chaloupka and Laixuthai 1997).

Two studies examined factors contributing to the mortality rate resulting from motor vehicle crashes among 18-to 20-year-olds between 1977 and 1992 (Dee 1999) or between 1977 and 1997 (Dee and Evans 2001). Both studies also reported significant negative effects of increases in the beer tax on the motor vehicle mortality rates. Dee (1999) and Dee and Evans (2001) dismiss these findings, however, because the researchers found similar tax effects regardless of whether they studied nighttime fatalities (which commonly are attributable to alcohol use) or daytime fatalities (which are related to alcohol use much less often). Yet one could argue that the potential pool of youth victims of fatal daytime crashes (i.e., youths who drink during the day and then drive), while smaller than the potential pool of victims of nighttime crashes, may be more sensitive to price than other youth drinkers. This would be the case if the youths in question are frequent or heavy drinkers, because as mentioned earlier, evidence suggests that those youths who drink frequently or heavily are quite sensitive to price.

Another study analyzed fatal motor vehicle crashes among people of all ages for the years 1984 to 1992 using fixed-effect models (Mast et al. 1999). These analyses found that the beer tax has no effect on the overall fatality rate but has a significant negative effect on the fatality rate for drivers involved in nighttime, single-vehicle crashes, which commonly involve alcohol. The investigators downplayed the importance of the beer tax, however, because the size of its effect varied when other variables were introduced into the models. Nevertheless, a careful examination of the study’s results reveals significant negative tax effects in most of the fixed-effects models used.^7^

The key conclusion to be drawn from this research is that increases in the full price of alcohol—whether they result from increases in monetary price, reduced availability, or increases in the expected legal costs of drinking and driving (i.e., more severe drunk-driving laws)—can reduce drinking and driving and its consequences among all age groups. As is the case with the effects of beer taxes on consumption, however, the estimated magnitude of the beer tax effects on motor vehicle mortality depends somewhat on whether State-fixed effects are included in the statistical models used. For example, Saffer and Grossman (1987*a*) reported much greater tax effects when using a fixed-effects model. Similarly, significant tax effects in the studies by Ruhm (1996), Dee (1999), Mast and colleagues (1999), and Dee and Evans (2001) also were based on this specification.

### Health Effects

Excessive alcohol consumption can have numerous adverse health effects; accordingly, reductions in alcohol consumption related to price increases might also reduce adverse health effects. Several studies have examined the impact of alcohol prices on liver cirrhosis mortality rates, a key adverse outcome associated with long-term heavy alcohol consumption that accounts for more than 20,000 deaths annually. For example, Cook and Tauchen (1982) analyzed annual State-level cirrhosis mortality rates for States that licensed the sale of alcoholic beverages from 1962 through 1977. The investigators concluded that increases in the excise taxes on distilled spirits would significantly reduce deaths from liver cirrhosis. For example, a $1 increase in the distilled spirits tax was estimated to lower cirrhosis death rates by 5.4 to 10.8 percent. Thus, the study contradicted the then-conventional wisdom that heavy, addictive alcohol consumption was unresponsive to price.

This finding was confirmed by Grossman (1993) when he applied the Becker-Murphy model of addiction to heavy alcohol consumption as reflected by the cirrhosis mortality rate. Using data for all States for the period from 1961 through 1984, Grossman concluded that long-term heavy consumption is responsive to price. For example, he estimated that a 10-percent increase in the price of alcohol would reduce cirrhosis mortality by 8.3 to 12.8 percent after the levels of heavy drinking have fully adjusted to the price change in future years. (This adjustment would extend over many years because due to the addictive nature of heavy drinking, a price increase in 1 year would reduce drinking not only in that year but also in all future years.)

In contrast to the two studies just discussed, Sloan and colleagues (1994), using State-level death rates for the period from 1982 through 1988, found that higher alcoholic beverage prices do not significantly reduce deaths that are primarily related to alcohol, mainly deaths from liver cirrhosis. This finding is surprising, given the results of the earlier studies. In addition, however, the study considered the effects of price on various other death rates related to alcohol use and abuse, including deaths from motor vehicle crashes, homicides (which are discussed in the following section), suicides, diseases for which alcohol is a contributing factor (e.g., cancers of the alimentary tract), and accidental deaths. Sloan and colleagues (1994) concluded that increases in the monetary price of alcoholic beverages would reduce suicides and deaths from diseases for which alcohol is a contributing factor, but not deaths that are primarily related to alcohol. Conversely, the study found that alcohol availability, which is another component of the full price of alcoholic beverages, has a significant impact on many of the death rates estimated, including deaths primarily related to alcohol; suicides; and deaths from drowning, falls, and other injuries.

Ohsfeldt and Morrisey (1997) also examined the impact of alcohol price and availability on injuries, specifically nonfatal workplace injuries, using State-level data for the period from 1975 through 1985. These analyses found a strong inverse relationship between workplace injuries and beer taxes. For example, the investigators predicted that a 25-cent increase in the beer tax in 1992 would have reduced work-loss days from nonfatal workplace injuries by 4.6 million, reducing the costs of lost productivity by $491 million. In contrast, alcohol availability has little impact on nonfatal workplace injuries according to these analyses.

Chesson and colleagues (2000) focused on a different outcome— sexually transmitted disease rates—in an analysis of all States for the years 1981 to 1995. After controlling for State and year effects, the investigators concluded that a $1 increase in the per-gallon liquor tax can reduce gonorrhea rates by 2.1 percent; furthermore, a beer tax increase of 20 cents per six-pack can reduce gonorrhea rates by 8.9 percent. Similar, or even somewhat larger, effects of liquor and beer taxes were found for syphilis rates.

Again, the general conclusion that can be drawn from this research is that increases in the full price of alcoholic beverages would reduce various diseases, injuries, and deaths related to alcohol use and abuse. Moreover, given the results of most studies analyzing liver cirrhosis rates, these reductions of adverse health effects would not be limited to injuries and deaths among light and moderate drinkers but would also affect heavy drinkers.

### Violence and Other Crime

Because a variety of crimes are related to alcohol use and abuse (Bureau of Justice Statistics 1988), numerous studies have assessed the influences of changes in alcohol prices on crime rates. For example, Cook and Moore (1993) examined the impact of per capita alcohol consumption and beer excise taxes on violent crime rates (i.e., homicides, assaults, rapes, and burglaries), using annual State-level data obtained from the 1979 through 1987 Uniform Crime Reports. Employing fixed-effects models, in which the only independent variable other than State and year indicators was the beer tax, the investigators concluded that higher beer taxes would lead to significant reductions in rapes and robberies but would have little impact on homicides and assaults. These findings are generally confirmed by an analysis of homicide rates obtained from the Vital Statistics data, which also concluded that higher alcoholic beverage prices and reduced alcohol availability would lower homicide rates (Sloan et al. 1994).

Several studies examined the effects of alcohol regulation on violence and crime using individual-level data. Markowitz and Grossman (1998) focused their analysis on child abuse, using data from the 1976 National Family Violence Survey on children residing in two-parent families. The study estimated the effects on violent outcomes of a variety of factors, including the State excise tax rate on beer, illegal drug prices, marijuana decriminalization, laws restricting alcohol advertising, per capita number of outlets licensed to sell alcohol, and demographic and socioeconomic characteristics of the parents. The results demonstrated that increases in the beer tax can be an effective policy tool in reducing child abuse. Thus, a 10-percent increase in the excise tax on beer was estimated to reduce the probabilities of overall child abuse and severe child abuse by 1.2 percent and 2.3 percent, respectively. Furthermore, such an increase was estimated to reduce unconditional overall child abuse (i.e., a measure of child abuse that includes the frequency of the abuse) by about 2.1 percent. Even such a seemingly small reduction in child abuse rates could have a dramatic impact on the lives of many children. In 1975, approximately 40 million children between the ages of 3 and 17 lived with both parents. According to the National Family Violence Survey, 14.4 percent of these children (i.e., 5.8 million) were victims of severe abuse. Hence, a 10-percent increase in the beer tax would have lowered the number of severely abused children by approximately 132,500.

Markowitz and Grossman (2000) expanded upon this study in two important ways. First, they performed the analyses separately by gender of the parent, which is important because different patterns of drinking and violence have been observed for men and women. Second, they added data from another comparable survey conducted 10 years later, the 1985 National Family Violence Survey, which allows for a comparison of the effects of alcohol regulation over time as well as for the pooling of several years and the addition of State-level fixed effects. Such fixed effects are important in determining whether the effects of the State-level alcohol regulation variables reflect State sentiment toward regulation and violence, which cannot be measured directly, rather than true policy effects.

The results of these analyses indicated that increases in the beer tax may decrease the incidence of child abuse committed by women but not by men. Thus, a 10-percent increase in the excise tax on beer was estimated to reduce the number of mothers who commit violent acts against their children by approximately 2 percent. This estimate was not influenced by State-level fixed effects, suggesting that it was indeed a policy effect.

Another study focused on the relationship between alcohol prices and spouse abuse (i.e., both wife abuse and husband abuse) (Markowitz 2000). This analysis used data from the 1985 National Family Violence Survey as well as respondents from that survey who were interviewed again in 1986 and 1987. Hence, the study was based on a panel of three observations on each person. The statistical analysis also included individual-level fixed effects to control for unmeasured characteristics in the panel. One example of such characteristics is the person’s sentiment toward alcohol consumption, which may be correlated with his or her propensity to commit violence, with overall alcohol sentiment in his or her State of residence, and with the rate at which alcohol is taxed. The results consistently indicated that increases in the price per ounce of pure alcohol (as measured by a weighted average of the prices of alcohol from beer, wine, and liquor) reduce the probability of severe violence (kicking, biting, hitting with a fist or other object, choking, and using or threatening to use a gun or knife) aimed at wives. Using an average of the estimates from the fixed-effects specification, Markowitz (2000) estimated that a 1-percent increase in the price per ounce of pure alcohol would decrease the probability of being a victim of wife abuse by 5.3 percent.^8^ This means that in 1985, when there were 54.4 million married women in the United States, of whom 3.6 percent were estimated to be abused, a 1-percent increase in the price of pure alcohol would have decreased the number of abused married women by approximately 104,600.

Grossman and Markowitz (2001) explored the effects of variations in alcoholic beverage prices among States on violence on college campuses. The study used data from the 1989, 1990, and 1991 Core Alcohol and Drug Surveys of College Students, which include almost 120,000 college students from approximately 200 colleges and universities throughout the United States and contain measures of alcohol use and its adverse consequences. These adverse consequences include four indicators of violence, as follows:

- Getting in trouble with the police or with residence hall or other college authorities
- Damaging property or pulling a fire alarm
- Getting into an argument or a fight
- Taking advantage of another person sexually or having been taken advantage of sexually.

The study found that the incidence of each of these four acts of violence is inversely related to the beer price in the State in which the student attends college. For example, a 10-percent price increase would result in the following reductions in violent acts:

- The proportion of students who get into trouble with the police and college authorities would decline from 12.3 percent to 11.7 percent.
- The proportion of students involved in property damage would be reduced from 7.5 percent to 7.1 percent.
- The percentage of students who get into verbal or physical fights would fall from 31.2 percent to 30.2 percent.
- The proportion of students involved in sexual misconduct would decline from 14.3 percent to 13.8 percent.
- The number of students involved in violence each year would be reduced by approximately 200,000, or by 4 percent.

Saffer (2001) estimated the effectiveness of alcohol and other drug abuse policies in reducing crime. The study used data from more than 32,000 people participating in the 1991 National Household Survey on Drug Abuse (NHSDA), which was complemented with data on State beer taxes. The analysis estimated the effectiveness of drug control spending and beer taxes on arrests, property crime, property damage, use of force, and drug selling, both for the entire sample and for people under age 21. The results demonstrated that increased beer taxes can reduce crime and that the magnitude of these effects generally is larger for people under age 21 than for people over age 21.

In summary, the findings discussed in this section clearly indicate that, as with alcohol consumption and other outcomes related to alcohol abuse, increases in the full price of alcoholic beverages are an effective means of reducing alcohol-related violence and other crime.

## Discussion

This article has summarized the economic research examining the impact of the full price of alcoholic beverages on drinking and heavy drinking by teenagers and young adults. It also has reviewed similar research that explores the relationship between price and outcomes related to the abuse of alcohol by youths and adults, including drinking and driving and motor vehicle crashes, health consequences of alcohol consumption, and violence and other crime. The majority of this research clearly supports the view that increases in the monetary prices of alcoholic beverages, which can be achieved by raising Federal, State and local alcohol taxes, significantly reduce alcohol consumption.

Of course, one must keep in mind the caveat mentioned previously concerning the need to exercise caution in interpreting cause-and-effect relationships from the types of analyses discussed in this article. Nevertheless, the weight of the evidence is impressive. Moreover, several studies have concluded that these reductions in consumption are not limited to the infrequent, light, or moderate drinkers but also pertain to frequent and heavy drinkers. Furthermore, increases in price also lead to reductions in many of the consequences of heavy drinking. Two studies, however, have suggested that a subset of heavy drinkers—the upper 5 percent—may be unresponsive to price (Manning et al. 1995; Kenkel 1996). Because both of these studies analyzed the drinking behavior of people of all ages, however, they are not inconsistent with the notion that youths and young adults—the age groups with disproportionately high alcohol-related problems—are generally more responsive to increases in price than are adults.

Given this evidence, increases in the prices of alcoholic beverages appear to be an effective policy for reducing alcohol consumption and its consequences. In reality, however, alcoholic beverage prices have declined relative to the prices of other goods and services for most of the past 50 years. This price decline is the result in large part of the infrequent and relatively small changes in Federal and State taxes. Based on the evidence presented here, it appears likely that this decline in real prices has kept alcohol consumption and many of the problems associated with alcohol use and abuse at levels higher than they would otherwise be.

In formulating the appropriate alcohol tax policies, it would be useful to have information on the differential price responsiveness of the outcomes considered here by gender, race, and ethnicity. For example, evidence suggests that certain drinking patterns are more sensitive to price among female college students than among male college students. It also appears as if child abuse committed by women is more responsive to price than child abuse committed by men, although that finding is based on fairly old data. To date, however, no large-scale studies have considered gender differences in price effects for a variety of outcomes in a systematic fashion. Similarly, little is known about racial or ethnic differences in price effects. Thus, researchers do not know whether the beneficial effects of tax increases on alcohol abuse will be shared equally by all population subgroups, or whether policies in addition to tax increases must be pursued to curtail abuse in certain groups.

Another area in which knowledge is lacking pertains to how the beneficial effects of moderate alcohol consumption would be altered by tax increases. For example, would coronary heart disease rise, and if so, by how much? And what is the trade-off between reductions in alcohol abuse and deteriorations in coronary health that may accompany tax hikes? Answers to these questions and the identification of population subgroups that are most or least sensitive to alcohol prices and taxes deserve high priority on an agenda for future research.

## Footnotes

1 These computations are based on Bureau of Labor Statistics data from various years.

2 For a definition of this and other economic terms used in this article, see the glossary, p. 32.

3 Leung and Phelps (1993) emphasize that these numbers represent “best guesses” because of the wide range of estimates contained in the studies reviewed.

4 For a detailed review of alternative economic models of addiction and their applicability to the estimation of the demand for alcoholic beverages and heavy drinking, see Grossman (1993).

5 These indicators were dichotomous variables for each State except one. This means that each variable assumes the value of 1 for a given State and 0 for all other States.

6 Omitted variables biases arise if determinants of fatal crashes are not included in the regression model and are correlated with variables used to predict these crashes.

7 The tax effect becomes insignificant in models accounting for the percentages of the State populations that are members of various religious denominations. This variable was available only for 2 of the years studied (i.e., 1980 and 1990), however, and had to be obtained for other years by interpolation and extrapolation.

8 Markowitz (2000) cautions that the value of this estimate is somewhat imprecise because the 95-percent confidence interval, which gives the range that with a 95-percent certainty contains the “real value” of the variable, is −1.0 to −9.7 percent.

## GLOSSARY

Aggregate data: Data based on information (e.g., on alcohol consumption) from large groups of people, such as the United States population as a whole or the population of individual States, either at a certain point in time or over time.
Dichotomous variable: Assumes the value of 1 if an observation has a certain characteristic and assumes the value of 0 if an observation does not have that characteristic; for example, a dichotomous variable for residence in California equals 1 if a consumer lives in California or if an observation on alcohol consumption or motor vehicle accident mortality pertains to California.
Economic model of addiction: A model in which current consumption of a certain good is positively related to past consumption of that good.
Farsighted model of addiction: A model in which consumers take into account the future harmful consequences of their current actions.
Fixed-effects regression model: Takes into account unmeasured effects that vary among States or individuals but do not vary over time by including *dichotomous variables* for States or individuals as independent variables; can be implemented only if there is more than one observation for each State or individual.
Individual-level data: Data based on information (e.g., on alcohol consumption) from specific people.
Long-term price elasticity of demand: Percentage change in current consumption resulting from a 1-percent increase in price several years earlier; exceeds *short-term price elasticity* in absolute value because a reduction in consumption this year resulting from an increase in price this year causes consumption to fall next year and in all future years.
Myopic model of addiction: A model in which consumers ignore the future harmful consequences of their current actions.
Price elasticity of demand: Percentage change in consumption resulting from a 1-percent increase in price.
Short-term price elasticity of demand: Percentage change in current consumption resulting from a 1-percent increase in price this year, with consumption last year held constant.
Utility maximizing approach: The approach to consumer behavior taken by economists in which consumers select the basket of goods and services that yields the largest amount of satisfaction or utility subject to their income and to the prices of those goods and services.
